# Supplementary figures and images for: A Bayesian approach to incorporate structural data into the mapping of genotype to antigenic phenotype of influenza A(H3N2) viruses
Source: PLoS Comput Biol. 2023 Mar 27;19(3):e1010885. doi: 10.1371/journal.pcbi.1010885 (PMC10079231; doi:10.1371/journal.pcbi.1010885)

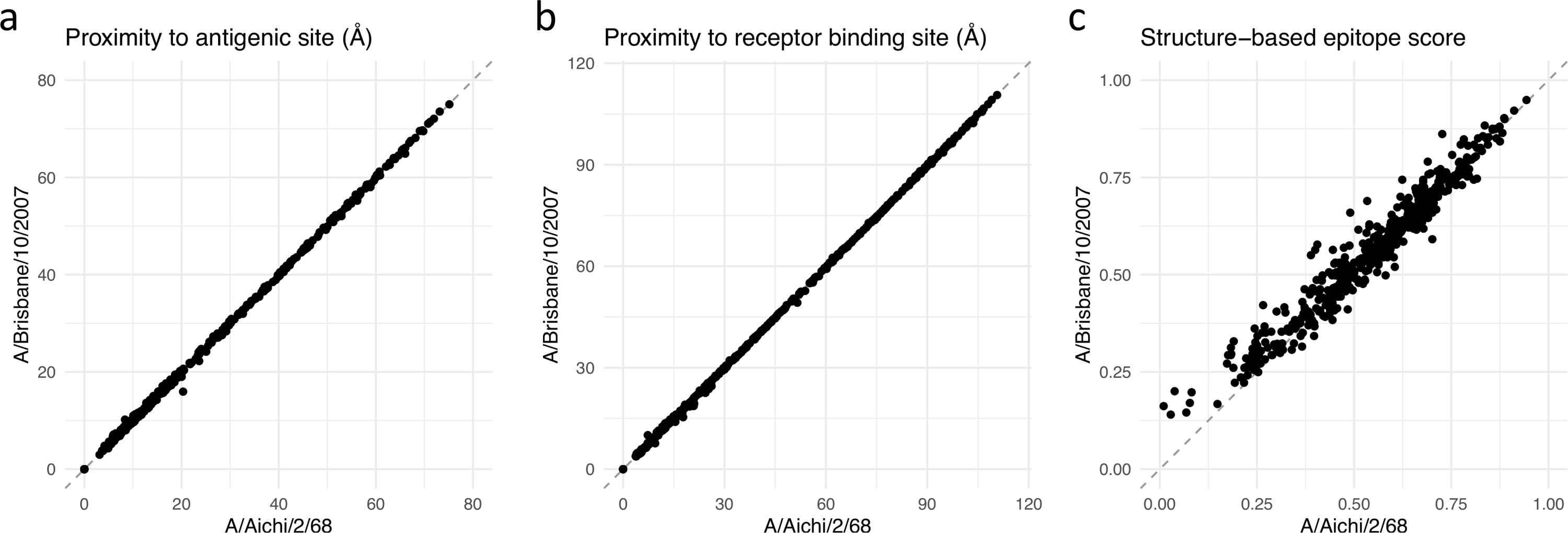

Supplement: S1 Fig — (a) The correlation in the distance of the alpha carbon of each HA residue to the closest alpha carbon of a residue belonging to a described antigenic site. (b) The correlation in the distance of the alpha carbon of each HA residue to the closest alpha carbon of a residue belonging to the receptor-binding site. (c) The correlation in structure-based epitope scores estimated for each HA residue using the software BEpro. (TIF) [file pcbi.1010885.s001.tif]

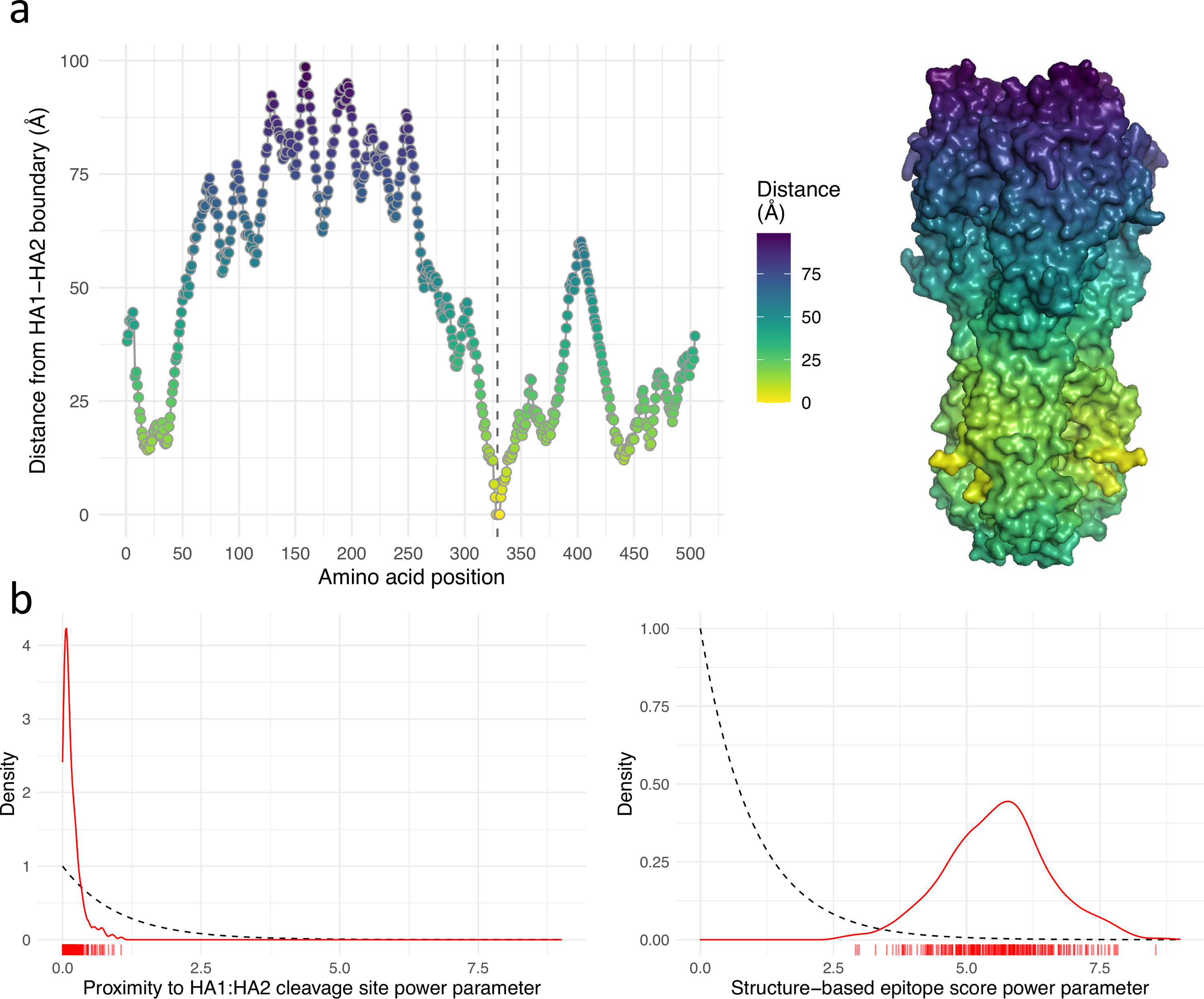

Supplement: S2 Fig — (a) The distance of each HA residue to the HA1-HA2 boundary where post-translational cleavage occurs. A vertical dashed line at position 329 indicates the boundary between HA1 and HA2. To the right, a surface representation of the HA is shown. (b) In each plot, posterior distributions for power terms that link proximity to the cleavage site (left) and structure-based epitope scores (right) for each HA position to a structure-informed probability for the position, π˘λ, according to Eq 8. Individual values sampled from the posterior distribution are shown below the x-axis. Prior distributions for these parameters, defined as Gamma(1, 1), are shown as dashed black lines. (TIF) [file pcbi.1010885.s002.tif]

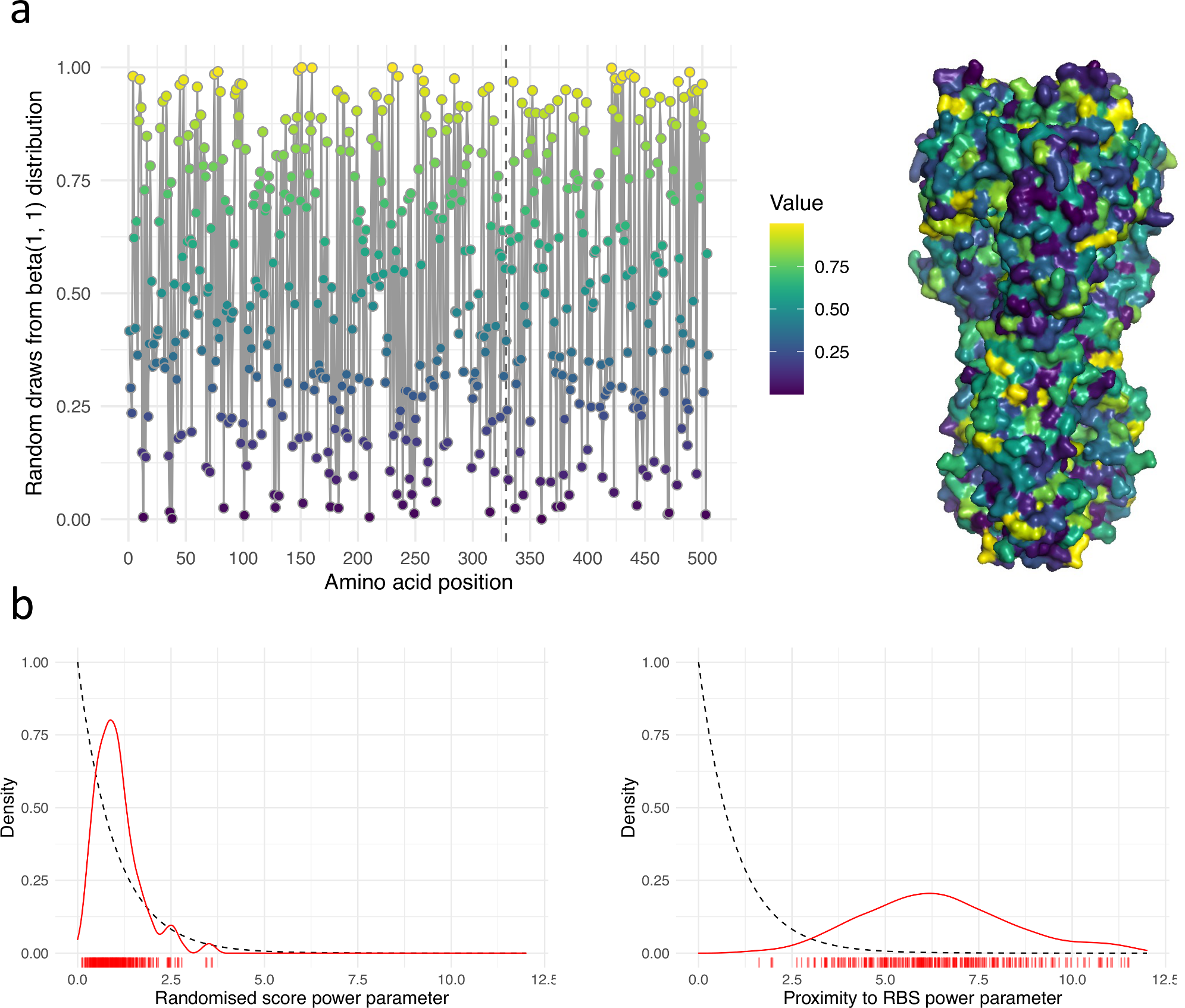

Supplement: S3 Fig — (a) Values randomly drawn from a beta(1,1) distribution and assigned to each HA residue. A vertical dashed line at position 329 indicates the boundary between HA1 and HA2. To the right, a surface representation of the HA is shown. (b) In each plot, posterior distributions for power terms that link randomly drawn values (left) and proximity to the RBS (right) for each HA position to a structure-informed probability for the position, π˘λ, according to Eq 8. Individual values sampled from the posterior distribution are shown below the x-axis. Prior distributions for these parameters, defined as Gamma(1, 1), are shown as dashed black lines. (TIF) [file pcbi.1010885.s003.tif]
