# Supplementary material for: A Bayesian approach to incorporate structural data into the mapping of genotype to antigenic phenotype of influenza A(H3N2) viruses
Source: PLoS Comput Biol. 2023 Mar 27;19(3):e1010885. doi: 10.1371/journal.pcbi.1010885 (PMC10079231; doi:10.1371/journal.pcbi.1010885)
Supplement: S1 Table — (DOCX) [file pcbi.1010885.s004.docx]

**Table S1. Model confidence in variable selection and distance of included or excluded HA positions to known antigenic sites.**

|  |  |  | Number of positions^a^ with given posterior inclusion probability and distance from antigenic site | | | |
| --- | --- | --- | --- | --- | --- | --- |
|  |  |  | Confidently included (Inclusion prob >.95) | | Confidently excluded (Inclusion prob <.05) | |
| Model | Symmetric substitution effects | Effective number of parameters^b^ | *N* positions | Mean distance (Å) | *N* positions | Mean distance (Å) |
| Phylogenetic model (**Eq 3**) | n/a | 63.6 (5.0) | *-* | - | *-* | - |
| Substitution model with independent effect sizes (**Eq 4**). | Yes | 70.1 (8.0) | 1 | 4.1 | 106 | 11.0 |
|  | No | 72.3 (8.7) | 1 | 5.2 | 135 | 10.8 |
| Substitution model with effect sizes linked to position (**Eq 5**). | Yes | 108.8 (7.6) | 6 | 0.9 | 113 | 12.3 |
|  | No | 127.8 (12.1) | 4 | 0.6 | 110 | 15.3 |
| Structure aware model – position dependent effect sizes for substitutions and structure influencing inclusion probability (**Eq 6**). | Yes | 128.9 (9.6) | 14 | 1.2 | 130 | 16.6 |
|  | No | 196.2 (14.0) | 16 | 1.0 | 91 | 20.9 |

^a^Total number of variable positions evaluated = 199. ^b^The effective number of explanatory variables, branch terms or substitutions, contributing significantly (> 0.1 log_2_ HI titre) to antigenic dissimilarity ($\Delta_{r,v}$ in **Eq 2**), measured in each MCMC sample and averaged across them (mean and standard deviation shown).
